# Road-to-Health Booklet: Insights from Vhembe district’s caregivers on health promotion practices

**DOI:** 10.4102/hsag.v30i0.3014

**Published:** 2025-10-10

**Authors:** Zwivhuya P. Mudau, Emmanuel Ramukakate, Tshifhiwa C. Mandiwana, Anzani Mugware, Lindelani F. Mushaphi, Selekane A. Motadi

**Affiliations:** 1 Department of Nutrition, Faculty of Health Sciences, University of Venda, Thohoyandou, South Africa

**Keywords:** caregivers, growth monitoring and promotion, knowledge, perception, road-to-health Booklet

## Abstract

**Background:**

Child growth monitoring and promotion give caregivers the knowledge and tools they need to track their children’s growth. The Road-to-Health Booklet (RtHB) is used to record the child’s health and development during clinic visits.

**Aim:**

The aim of this study is to explore caregivers’ use of the RtHB in selected villages within Thulamela Municipality.

**Setting:**

The study was conducted in selected villages within Thulamela Municipality.

**Methods:**

A qualitative descriptive exploratory study was conducted using a semi-structured interview guide to gain insights into caregivers’ experiences with the RtHB. Purposive sampling was used to select 28 caregivers of children under 5 years of age for this research. In-depth interviews were conducted to gather comprehensive data, which were recorded through field notes and an audio recorder. Thematic content analysis was employed to analyse the collected data.

**Results:**

Findings revealed that caregivers perceive the RtHB as a critical tool provided after delivery for maintaining records of their children’s health status, growth and immunisation history. Some caregivers indicated that some of the information should be excluded from the Booklet because they believe information such as HIV status is confidential.

**Conclusion:**

In Thulamela municipality, caregivers demonstrated a clear understanding of the RtHB, including its purpose, appropriate usage and benefits. However, some expressed concern about the inclusion of HIV status in the Booklet. There is a need for the Department of Health to come up with strategies to keep confidential information such as HIV status through the use of code on the Booklet.

**Contribution:**

The results may contribute to the body of literature related to the caregivers’ use of the RtHB.

## Introduction

Undernutrition is the leading cause of 45% of all childhood deaths, which mostly occur in low- and middle-income countries (Madewell et al. 2024). Undernutrition during childhood has a substantial negative impact on a child’s growth and development (Ronaasen et al. 2018). Furthermore, the first 5 years of a child’s life are critical for growth and development. Undernutrition can be prevented during childhood through proper growth monitoring (Mabesa, Knight & Nkwanyana 2023). Child Growth Monitoring and Promotion (GMP) equips mothers and caregivers with a simple tool to track their children’s growth and development (Sibanda, Mbhenyane & Mushaphi 2016). Growth Monitoring and Promotion is one of the intervention strategies that play a vital role in reducing infant mortality, improving nutritional status and increasing the use of health services worldwide (Hawkes et al. 2020; Taylor et al. 2023).

The Road-to-Health Booklet (RtHB) is a tool used to monitor the growth of children under 5 years of age (Naidoo, Avenant & Goga 2018). Introduced in South Africa in 1973, it has played a significant role in child health promotion and guiding early interventions (Naidoo et al. 2018). The National Department of Health in South Africa distributes the RtHB to caregivers, helping healthcare workers monitor children’s development and communicate relevant health messages (DoH 2021). The RtHB is updated at every clinical visit to track the child’s growth and health. According to Naidoo et al. (2018), the RtHB serves as an essential patient-held medical record, summarising a child’s health during their first 5 years of life. In addition, it provides healthcare workers and caregivers with essential information about the child’s growth trends (Blaauw et al. 2017). The RtHB is designed to be a simple, cost-effective and practical method for monitoring growth and development, promoting child well-being and allowing for the early detection of health issues so that timely intervention strategies can be implemented (Galasso et al. 2019).

Several studies have been conducted in various provinces in South Africa that assess the knowledge of healthcare workers on the RtHB and their interpretation of anthropometric measurements (Blaauw et al. 2017; Legoale & Manafe 2024; Mabesa et al. 2023; Mangena 2020). Some studies focused on the implementation and utilisation of the South African RtHB and included challenges experienced by health professionals (Machimana et al. 2024; Mangena 2020; Win & Mlambo 2020). A study conducted by Win and Mlambo (2020) reported several issues, such as caregivers forgetting to bring the Booklet to clinic visits, tearing out HIV-related pages, missing appointments, or losing the Booklet altogether, disrupting the tracking of immunisation records and contributing to increased child morbidity and mortality rates. At the time of conducting this study, no studies have been conducted on caregivers’ use of the RtHB in Thulamela municipality. In light of this, this study aims to explore the use of the RtHB by caregivers in selected villages within Thulamela Municipality, Vhembe District, Limpopo province, South Africa.

## Research methods and design

### Research design

A qualitative approach, utilising exploratory and descriptive methods, was employed in this study to examine the use of the RtHB by caregivers in two selected semi-rural villages in Thulamela Municipality, namely Muledane and Shayandima villages. This approach was chosen because it allows for an in-depth exploration of caregivers’ experiences, perceptions and challenges in using the RtHB (Taherdoost 2022).

### Study setting

The study was conducted in Thulamela Municipality. The Municipality is one of the four municipalities located within the Vhembe District, which is the second-smallest municipality in the district. It has a population of approximately 618 462 and an unemployment rate of 58.3%. Notably, 4.1% of residents reported being too poor to afford food, the highest percentage compared to other municipalities in the area (Stats SA 2022). The municipality has 49 clinics, three Primary Health Centres (PHC) and two district hospitals (South Africa National Census 2022). The municipality also recorded a mere 4.4% PHC utilisation rate for children under 5 years of age. In Vhembe district, the leading causes of death include severe acute malnutrition (15.5%) and diarrhoeal diseases (19.3%) affecting populations across all four municipalities (Massyn et al. 2020). The Thulamela Municipality was chosen because of the persistent undernutrition concern among children under 5 years (Mugware et al. 2022). The study was conducted in two selected semi-rural villages, namely Muledane and Shayandima, each of which has its own health care facility.

### Study population and sampling

The target population for this study consisted of caregivers of children under 5 years of age from two selected semi-rural villages in Thulamela Municipality. The caregivers were selected using the purposive sampling technique. The sample size for the qualitative study was determined by data saturation. The data saturation was attained at 24 caregivers and four additional caregivers were added to see if any new information appeared. No additional insights emerged beyond caregiver 28. The researcher, with prior knowledge of households with children under five, collaborated with royal advisors (courtiers and community leaders) in the selected villages to identify additional households. Recruitment took place at community gatherings held at the chief’s kraal (royal house), and researchers also visited selected caregivers at their homes to explain the study’s aims, objectives and procedures. Caregivers with children under 5 years of age who agreed to participate and signed informed consent forms before the study commenced were included. All the caregivers who are health care professionals were excluded from the study to reduce the bias. Interviews were then conducted at the caregivers’ homes during follow-up visits.

### Data collection

The data were collected by the researcher and two trained research assistants. The interview guide was used to collect data. The interview guide was pre-tested and piloted prior to data collection. After the pre-testing and piloting, no amendment was made to the instrument. The socio-demographic information of the caregivers, such as age, occupation, level of education and marital status, was collected and noted. In-depth interviews were conducted to collect data using an interview guide with one main open-ended question: ‘What do the caregivers understand about the use of the RtHB?’ Additional questions were generated through probing. All the interview sessions were recorded using a voice recorder with permission from the caregiver. Field notes were taken during the interviews. Interviews were conducted in a quiet setting within the caregivers’ homes to ensure privacy. The interviews were conducted using the local language (Tshivenda). The interviews were conducted by the researcher with two assistants. Each interview lasted approximately 30 min – 60 min (Holloway & Galvin 2023).

### Data analysis

Thematic content analysis was used for data analysis. Thematic content analysis follows six steps and involves three key stages: data reduction, data display and conclusion drawing (Lyons & Colyle 2016). The qualitative data from the interviews were analysed using both text and content analysis. Interviews were transcribed and translated into English with the assistance of a language practitioner from the University of Venda. The researcher read all the transcriptions repeatedly to give the segment data meaning. The notebook was used to record the meanings and insights that emerged while reading the verbatim transcripts, along with related thoughts. Throughout this process, similar topics were grouped together, and dissimilar ones were clustered separately. The researcher created abbreviations for the emerging topics, writing these codes next to the relevant segments of the transcription. Themes and subthemes were developed from the coded data and the associated texts. The researcher then reviewed the entire work from the beginning to check for duplications and refined the codes, topics and themes as needed. An independent coder also collaborated with the researcher to confirm the identified themes and subthemes before the final research was produced.

### Trustworthiness

The researcher ensured the trustworthiness of the study by applying the principles of credibility, confirmability, dependability and transferability (Ahmed 2024; Stahl & King 2020). To ensure credibility, the researcher conducted the interviews to ensure sufficient data collection until saturation occurs. Semi-structured individual interviews were conducted over 2 weeks, with each interview lasting up to 30 min – 60 min, continuing until data saturation was achieved (Holloway & Galvin 2023). Confirmability was maintained by using an audio recorder to capture participants’ responses in their original, unaltered form (Ahmed 2024). To ensure transferability, the researcher thoroughly described the context of the study, including an accurate description of the participants, the sampling process and the date, time and location of data collection (Ahmed 2024). Dependability was ensured through triangulation, where two researchers independently interviewed participants and separately processed and analysed data from different villages, ensuring consistency and reliability in the information collected.

### Ethical considerations

The study received ethical clearance from the University of Venda Human and Clinical Trials Research Ethics Committee (SHS/19/NUT/06/1109). The study was conducted in accordance with the principles of the Declaration of Helsinki, protecting the dignity and rights of the participants, good clinical practices and the laws of South Africa. Before the study began, mothers received both oral and written explanations outlining its purpose and procedures. The caregivers were given an informed consent form to sign before participation. The caregivers were also informed about the right to withdraw from the study at any time without any repercussions.

## Results

The demographic data of 28 caregivers are summarised in terms of age, number of children, education, occupation and marital status (Table 1). More than half (53.6%, *n* = 15) of the caregivers were between the age of 20 and 29, 28.6% (*n* = 8) were between the ages of 30 and 39 years, 10.7% (*n* = 3) were between the ages of 40 and 49 years, while 7.1% (*n* = 2) were between the ages of 50 and 59 years. In terms of children, 35.7% (*n* = 10) had one child, 28.6% (*n* = 8) had two children, 17.9% (*n* = 5) had three, 10.7% (*n* = 3) had four and 7.1% (*n* = 2) had five children. Regarding educational attainment, 60.7% (*n* = 17) had completed high school (matriculated), while 39.3% (*n* = 10) pursued tertiary education. Most caregivers (78.6%, *n* = 22) were unemployed, with only 21.4% (*n* = 6) employed. Marital status data revealed that 78.6% (*n* = 22) were unmarried, 17.9% (*n* = 5) were married and 3.6% (*n* = 1) were widowed.

**TABLE 1:** Demographic profile of caregivers (*N* = 28).

Variables	*n*	Percentage
**Age range (years)**		
20–29	15	53.6
30–39	8	28.6
40–49	3	10.7
50–59	2	7.1
**Gender**		
Female	28	100.0
Male	0	0.0
**Number of children**		
One	10	35.7
Two	8	28.6
Three	5	17.9
Four	3	10.7
Five	2	7.1
**Highest qualification**		
Secondary level	17	60.7
Tertiary level	11	39.3
**Occupation**		
Employed	6	21.4
Unemployed	22	78.6
**Marital status**		
Single	22	78.6
Married	5	17.9
Widowed	1	3.6

From the interviews, there was one theme and four sub-themes that emanated from probing questions. Table 2 provides the theme and sub-themes that emerged from the data.

**TABLE 2:** Emerging theme and sub-themes from caregivers’ understanding about the use of the Road-to-Health Booklet.

Themes	Sub-themes
1. Caregivers’ understanding about the use of the RtHB	1.1. The use of the RtHB 1.2. Caregivers’ understanding on the benefits of RtHB use 1.3. Caregiver’s perceptions on the use of the RtHB 1.4. Caregivers’ roles on the use of the RtHB

RtHB, Road-to-Health Booklet.

### Theme 1: Caregivers’ understanding of the use of the Road-to-Health Booklet

The study revealed that caregivers perceive the RtHB as a tool provided after childbirth, which helps them maintain a record of their child’s health and development from birth through early childhood. The Booklet captures important information such as parental details, the child’s date of birth, weight, height, immunisations and records of clinic visits, whether for illness or routine check-ups.

#### Sub-theme 1.1: The use of the Road-to-Health Booklet

Caregivers highlighted that the RtHB is essential for tracking a child’s growth, recording illnesses and monitoring immunisations. They also referred to the Booklet at different developmental stages of the child. It is used during every healthcare visit, whether for medical treatment or routine immunisations. The Booklet is used to ensure that the child’s immunisations, growth and health milestones are accurately documented and tracked. This is supported by the following statements:

‘We are talking about the booklet that is given to the mother after birth. It serves as a record of all health-related information since the birth of the child including date of birth, height, and weight scaling, whether the child cried after birth, did he or she get vaccinated? The booklet records important health information which helps both parents and caregivers to monitor children’s schedules. Also, it gives records of all injections a doctor injects to a child.’ (Caregiver 1, age 32, unemployed)

‘The booklet is used to record the child’s health status, and which vaccines and drops are supposedly offered to a child based on the child’s age. The booklet works always when the child visits a health care institution.’ (Caregiver, age 29, employed)

Some caregivers expressed varying opinions regarding the use of the RtHB for both infants and young children, as well as for themselves as caregivers:

‘Booklet is used to records all injections that the child has received since birth and shows all the appointments to be followed, children’s growth, whether the child has malnutrition problems, whether the child is able to sit on time, his or her weight increasing or decreasing.’ (Caregiver 1, age 32, unemployed)

Caregivers highlighted that RtHB should be presented during every visit to a health facility, regardless of whether the child is sick or attending for growth monitoring and immunisation. However, some caregivers had differing perspectives on when the Booklet is most essential during these visits. This is supported by the following statements:

‘I think the booklet is used mainly to record all drops of medication and injections the child has received.’ (Caregiver, age 28, unemployed)

Another caregiver said, ‘I understand the booklet as a tool to get health services and that one cannot get child health services without the booklet’ (Caregiver 12, age 37, unemployed).

Additional perspective from one of the caregivers was ‘The road to health booklet gives access to health services monthly in order for the child to access the necessary health services freely’ (Caregiver 12, age 37, unemployed).

Caregivers reported that the use of the Booklets is to keep a record of the child’s growth and all the health-related information. Furthermore, the caregivers indicated that the Booklet should not be used to keep some of the information, such as HIV status. This is supported by the following statements:

‘The booklet should be taken to clinic only when the child visits Growth monitoring and promotion [*GMP*]. Some of the information in the booklet should be excluded as the booklets are also submitted at early childhood development centers [*ECDs*], and it is confidential information such as HIV and AIDS status.’ (Caregiver 13, age 34, unemployed)

#### Sub-theme 1.2: Caregivers’ understanding of the benefits of the use of Road-to-Health Booklet

In response to whether caregivers know the benefits of RtHB use, some caregivers expressed positive responses to caregivers, whereas others only knew that the Booklet is beneficial to the child only. The following statements support caregivers’ understanding of the benefits of using the RtHB:

‘I know the booklet offers me guidance in relation to the child’s medical status, including weight and height measurements, drops, injections and medicines a child must be given based on his and her wellbeing. Parents get informed of the medical attention the child needs.’ (Caregiver 3, age 25, unemployed and Caregiver 12, age 37, unemployed)

‘As parents, we are pleased to be given such booklet as it helps to record a child medical history when visiting a clinic.’ (Caregiver 1, age 32, unemployed)

Caregivers highlighted that the RtHB provides benefits for both them and their children. It keeps caregivers informed about their child’s developmental stages, medical history, immunisations and the weight and height measurements recorded at each visit. In addition, it outlines any interventions taken if the child experiences complications. For the child, the Booklet ensures they receive all necessary vaccinations, which help protect them from various diseases. This is supported by the following statements:

‘Benefits are shared to both mother and child, as the mother gets to know the child’s growth rate and when the child has grown enough, he or she can be showed his or her records. The child benefits by receiving all services the booklet states that the child must receive such as immunization and weight or height measurements that shows if the child is growing well or not, if not what intervention should be taken.’ (Caregiver 8, age 42, employed)

‘The mother is offered the benefit of accessing all health records in a detailed manner.’ (Caregiver 9, age 51, unemployed)

‘The main benefit is that children are given health care services that promote their proper growth and development.’ (Caregiver 10, age 27, unemployed)

#### Sub-theme 1.3: Caregivers’ perceptions on the use of Road-to-Health Booklet

The RtHB is regarded as a valuable tool for recording a child’s growth, medical history and developmental milestones. Caregivers view the Booklet as an important resource that should be kept safe, as it enhances their understanding of their children’s progress. This is supported by the following statement:

‘The road to health booklet is of much importance and has to be kept safe as it helps us assess the child’s growth and mental being as the child grows.’ (Caregiver 4, age 33, employed)

Caregivers perceive that the Booklet is made to be used by both caregivers and nurses. They refer to the Booklet when their children have complications and if needs be, they involve nurses to assist them. This is supported by the following statement:

‘It is easier to know the faults and schedules a child goes through with accurate reports and all vaccines to be given, when anything goes wrong the booklet works as a guide on what to do next then consult nurses if necessary.’ (Caregiver 14, age 27, unemployed)

#### Sub-theme 1.4: Caregivers’ roles on the use of the Road-to-Health Booklet

The role of a caregiver in the use of the RtHB is to keep and store the Booklet in a safe and accessible place; even when they are travelling with their children, they should always have it so that it is easier to help the child in case of emergencies. This is supported by the following statement:

‘The booklet must be kept safe and make sure it doesn’t get lost or damaged since all health information is recorded on the road to health booklet of a particular child. The Booklet holds all records of children’s weight, height, and all drops given.’ (Caregiver 1, age 32, unemployed and Caregiver 12, age 37, unemployed)

All the caregivers in the study understand that their role in the use of the Booklet is to keep it safe as well as present it to the health care facility each time they visit. They should always ensure they present the Booklet in every health care institution visit and ensure that all services offered to the child are recorded in the Booklet. This is supported by the following statement:

‘The safety of the booklet is the responsibility of the caregiver. She must keep it safe until the child has grown and provide the booklet in every clinic she goes to with the child.’ (Caregivers 6, age 31, unemployed)

## Discussion

This study suggests that caregivers demonstrated a strong understanding of the RtHB’s purpose and function in monitoring child health. This high level of understanding may be attributed to the high education levels observed among caregivers in the study. Education is a key factor influencing health literacy, including the ability to interpret and use health tools such as growth monitoring charts and immunisation records. The high level of understanding of RtHB usage may also be attributed to the fact that caregivers receive regular education about the Booklet during growth monitoring visits at clinics and health centres (Marume, Mahomed & Archary 2022). A strong understanding of the uses and the benefits of RtHB enables caregivers to identify potential health issues early, allowing for prompt interventions. These findings are consistent with those reported by Mangena (2020) and George (2021), who observed that caregivers with higher education levels were more likely to correctly use the RtHB and actively track their children’s immunisation and growth compared to those with lower educational attainment. Maternal education has been shown to significantly impact child health outcomes by improving caregivers’ ability to understand health information and adhere to preventive care practices (Mengistu et al. 2025; Mensch et al. 2019).

In this study, caregivers perceived the RtHB as a comprehensive tool for tracking their child’s journey from birth to adulthood, documenting all significant events and milestones. They found that that it maintains essential records, including parental details, the child’s date of birth, weight, height, immunisations and all clinic visits, whether for medical treatment or vaccinations. Caregivers also remarked that the RtHB aids in reporting their child’s developmental stage. The findings of this study are consistent with those of a study conducted by Mangena (2020) in the Ekurhuleni Metropolitan Municipality, Gauteng, which reported that caregivers viewed the RtHB as a valuable tool for tracking children’s health from birth to adulthood and for improving the health of infants and young children.

The results of this research highlighted that the primary purpose of the RtHB is to provide visible documentation of a child’s growth, enabling both healthcare workers and caregivers to monitor and improve the child’s nutritional status. These findings are consistent with existing literature that positions the RtHB not merely as a record-keeping tool, but as an interactive communication platform between caregivers and the health system (DoH 2021). The National Department of Health (2021) emphasised that the RtHB was designed to strengthen continuity of care and actively engage caregivers in the promotion of their children’s health. When effectively used, the RtHB contributes to improved health-seeking behaviours and enhances caregivers’ understanding of nutritional and developmental milestones (Du Plessis et al. 2017). Moreover, the visibility of growth data provided by the RtHB plays a crucial role in the early detection of growth faltering and in guiding the development of appropriate intervention strategies (Taylor et al. 2023). These findings further align with Machimana et al. (2024), who highlighted the RtHB as a key instrument in growth monitoring, facilitating nutritional assessments and supporting informed clinical decisions regarding a child’s development. For the RtHB to function optimally as both an educational and screening tool, it is essential that healthcare workers are adequately trained in its use and interpretation.

In this study, the caregivers reported other functions of the RtHB besides growth monitoring, such as the HIV status of the mother. These findings are in line with reports from Win and Mlambo (2020) and Machimana et al. (2024), who noticed that the Booklet serves as a convenient means of monitoring child health and nutritional status, while also providing essential health for the mother, such as HIV status. According to Win and Mlambo (2020), the RtHB contains vital health information, including immunisation records, head circumference, developmental screenings, vitamin A supplementation, prevention of mother-to-child transmission (PMTCT), deworming medications and oral health records. In addition, the RtHB is considered a cornerstone of primary health care and provides a helpful summary of a child’s health during the first 5 years, playing a crucial role in preventing child mortality and morbidity.

This study indicated that both caregivers and children benefit from using the RtHB. Caregivers recognised that the Booklet empowers them to take responsibility for their child’s health by keeping track of growth monitoring appointments, immunisations and medications administered. This observation corresponds with other authors who observed that the Booklet aims to ensure young children receive comprehensive developmental care services, both at health facilities and at home (Machimana et al. 2024; Mlambo 2020). By recording growth, immunisations and health interventions, the RtHB serves as a vital source of information for caregivers and fosters collaboration between them and healthcare workers. Caregivers are encouraged to present the Booklet at every healthcare visit, while healthcare workers provide support and advice regarding children’s health (Marume et al. 2022).

In 2021, the Department of Health recommended that caregivers must always bring the RtHB along every time a child visits a healthcare facility (DoH 2021). In this study, caregivers recognised their responsibility to bring the RtHB to each clinic visit and ensure its safekeeping. In addition, caregivers understood the importance of having their child assessed and weighed during every immunisation and consultation visit, with the results recorded in the RtHB. This underscores the Booklet’s role in continuous health monitoring and the active participation of caregivers in their child’s healthcare. The findings of this study aligned with those of Mabesa et al. (2023), where most caregivers understood that in every clinic visit, RtHB is used for immunisations, recording a child’s health, recording illness and taking a child to the clinic. The education caregivers receive at clinics and health centres significantly impacts their understanding of the RtHB and their roles as caregivers. Thus, the Booklet remains an essential tool for improving the health of infants and young children, aligning with the Sustainable Development Goals and the Global Strategy for the health of women, children and adolescents.

### Strengths and limitations

This study offered a more detailed understanding of the use of the RtHB by the caregivers with children under 5 years. In addition, this research focuses on under-researched areas, and this could benefit the Department of Health in terms of implementing intervention strategies to address malnutrition among children under five. This study was limited to the Thulamela Municipality in the Venda region; it is possible that its findings do not accurately represent behaviours across the provinces of Limpopo and South Africa. The anthropometric assessment was not performed during the data collection. Therefore, the growth and development of children were not assessed. Mothers were not assessed on the interpretation of the RtHB charts.

## Conclusion and recommendations

In Thulamela municipality, caregivers demonstrated a clear understanding of the RtHB, including its purpose, appropriate usage and benefits. The caregivers reported that the Booklet records important health information, which helps both parents and caregivers to monitor children’s schedules. Furthermore, the study highlighted how RtHB fosters collaboration between healthcare workers and caregivers, as caregivers are encouraged to present the Booklet during each healthcare visit. However, some caregivers indicated that some of the information should be excluded from the Booklet because they believe information such as HIV status is confidential. Although most of the caregivers demonstrated high knowledge regarding the use of the RtHB in selected villages within Thulamela Municipality, there is a need for awareness campaign initiatives in the community to emphasise the importance of the use of the RtHB amongst caregivers. There is a need for the Department of Health to come up with strategies to keep confidential information such as HIV status, which may be compromised due to the inclusion of individual names on the Booklet. The use of a code system within the Booklet could enhance confidentiality. The department should consider introducing a digital version of the Booklet, particularly for younger caregivers, as it offers convenience and accessibility.

### Primary contribution of the study

This study contributes to the existing literature by highlighting that caregivers possess a clear understanding of the RtHB, including its purpose, appropriate usage, benefits and their responsibilities in utilising it. The results may contribute to the body of literature related to the caregivers’ use of the RtHB. This study has the potential to improve the health status of children under five.
